# Crystallographic interface control of the plasmonic photocatalyst consisting of gold nanoparticles and titanium(iv) oxide†

**DOI:** 10.1039/d2sc03549a

**Published:** 2022-10-14

**Authors:** Shin-ichi Naya, Atsunobu Akita, Yoko Morita, Musashi Fujishima, Hiroaki Tada

**Affiliations:** Environmental Research Laboratory, Kindai University 3-4-1, Kowakae Higashi-Osaka 577-8502 Osaka Japan; Graduate School of Science and Engineering, Kindai University 3-4-1, Kowakae Higashi-Osaka 577-8502 Osaka Japan h-tada@apch.kindai.ac.jp; Department of Applied Chemistry, Faculty of Science and Engineering, Kindai University 3-4-1, Kowakae Higashi-Osaka 577-8502 Osaka Japan

## Abstract

A big question in the field of plasmonic photocatalysis is why a typical photocatalyst consisting of gold nanoparticles and rutile titanium(iv) oxide (Au/R-TiO_2_) usually exhibits activity much higher than that of Au/anatase TiO_2_ (Au/A-TiO_2_) under visible-light irradiation. Shedding light on the origin should present important guidelines for the material design of plasmonic photocatalysts. Au nanoparticles (NPs) were loaded on ordinary irregular-shaped TiO_2_ particles by the conventional deposition precipitation method. Transmission electron microscopy analyses for the Au/TiO_2_ particles ascertain that faceting of Au NPs is induced on R-TiO_2_ by using a domain-matching epitaxial junction with the orientation of (111)_Au_//(110)_R-TiO_2__, whereas non-faceted hemispherical Au NPs are exclusively formed on A-TiO_2_. The faceting probability of Au NPs (*P*_f_) on R-TiO_2_ increases with decreasing Au particle size (*d*_Au_) to reach 14% at *d*_Au_ = 3.6 nm. A clear positive correlation between the photocatalytic activity and *P*_f_ in several test reactions indicates that the heteroepitaxial junction-induced faceting of Au NPs is the principal factor for governing the plasmonic photocatalytic activity of Au/TiO_2_. In light of this finding, R-TiO_2_ nanorods with a high percentage (95%) of {110} facets were hydrothermally synthesized and used for the support of Au NPs. Consequently, the *P*_f_ value increases to as much as 94% to enhance the photocatalytic activity with respect to that of Au/R-TiO_2_ with *P*_f_ = 14% by factors of 2.2–4.4 depending on the type of reaction.

## Abstract 2

In the represented plasmonic photocatalyst consisting of Au nanoparticles (NPs) and TiO_2_, the combination of crystal facet engineering of TiO_2_ and atom-level-interface control between Au NP and TiO_2_ gives rise to a drastic activity enhancement.

## Graphical abstract

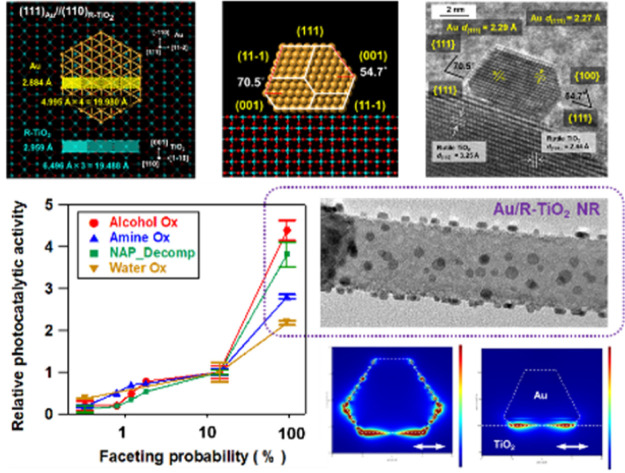

## Introduction

Photocatalysts are the key materials for the solar-to-chemical transformations, and their development is pivotal for dealing with increasing global energy demands and environmental issues. While research on semiconductor photocatalysts has been ongoing for many years, gold nanoparticle (NP) based-plasmonic photocatalysts represented by Au/TiO_2_ have recently attracted much attention as a new type of visible-light responsive photocatalysts.^1–6^ According to the action mechanism, plasmonic photocatalysts consisting of Au NPs and semiconductors can be classified into the hot-electron transfer (HET) type and local electric field enhancement (LEFE) or plasmon-resonance energy transfer type.^7^ The former possesses an outstanding feature that photons with the energy below the band gap of the semiconductor can be utilized as the energy source for chemical reactions,^8,9^ whereas the available photons are basically limited to the ones with the energy above the band gap in the latter.^10^ HET-type plasmonic photocatalysis is initiated by the excitation of the localized surface plasmon resonance (LSPR) of Au NPs and/or the interband transition (light harvesting or hot-carrier generation). Part of the hot electrons generated in the Au NP are injected into the conduction band (CB) of the semiconductor (hot electron injection).^11^ The CB-electrons are kept away from the holes in the Au NPs by using the Schottky junction between Au NPs and the semiconductor (charge separation).^12^ Consequently, the electrons escaping from recombination are used for reduction on the semiconductor surface, while the holes in the Au NPs cause oxidation on the Au surface or the interface with the semiconductor (surface chemical reactions). So far, much effort has been devoted to increase the activity of HET-type plasmonic photocatalysts by separately enhancing each physical and chemical process through the control of the size and shape of Au NPs,^13^ and the assembled structure.^14^ In contrast, the effect of the semiconductor on the activity is not fully understood, and research on the Au NP-semiconductor interface is still in its infancy.^15^ In this study, we focus on an unresolved issue that Au/rutile TiO_2_ (Au/R-TiO_2_) exhibits plasmonic photocatalytic activity much higher than that of Au/anatase TiO_2_ (Au/A-TiO_2_) for various reactions (Table S1†) in contrast to the UV-light activity of R-TiO_2_, which is lower than that of A-TiO_2_.^16^ Among the reactions, the oxygen evolution reaction (OER) has a large activation energy and is usually the bottle neck in the artificial photosynthetic reactions.^17^ Recently, Au/R-TiO_2_ has also been reported to show much higher plasmonic photocatalytic activity than Au/A-TiO_2_.^18^ Density functional theory calculations for a Au_8_/TiO_2_ model suggested that the rate-determining step of the plasmonic OER is the formation of *OOH, and the activation energy for the process in the Au/R-TiO_2_ system is lower than that in the Au/A-TiO_2_ system, where the symbol * expresses the adatom. However, the general superiority of Au/R-TiO_2_ to Au/A-TiO_2_ in the HET-induced plasmonic reactions suggests that the TiO_2_ crystal form mainly affects the physical processes involving light harvesting, hot electron injection, and charge separation rather than the chemical reaction process. The understanding of this origin would provide important and versatile guidelines for material design because the physical processes are common to the plasmonic photocatalytic reactions.

## Experimental

### Materials

Hydrogen tetrachloroaurate(III) tetrahydrate (HAuCl_4_·4H_2_O > 99%), urea (CH_4_N_2_O > 99.0%), benzylamine (C_7_H_9_N >98.0%), and 2-naphthol (C_10_H_8_O > 99.0%) were purchased from Kanto Chemical Co. Cinnamyl alcohol (C_9_H_10_O > 97.0%) was purchased from Tokyo Chemical Industry Co. Titanium bis(ammonium lactate) dihydroxide ([CH_3_CH(O^−^)CO_2_NH_4_]_2_Ti(OH)_2_, 50 wt% in H_2_O) solution was purchased from Sigma-Aldrich Co. Irregular-shaped anatase TiO_2_ particles (mean particle size = 150 nm, specific surface area = 8.1 m^2^ g^−1^, A-100, Ishihara Sangyo) and rutile-TiO_2_ particles (mean particle size = 80 nm, specific surface area = 18 m^2^ g^−1^, MT-700B, TAYCA) were used as the TiO_2_ supports. All chemicals were used as received without further purification.

### Rutile TiO_2_ nanorod synthesis

Rutile TiO_2_ NRs with {110} side walls were synthesized by the hydrothermal method.^19^ Titanium bis(ammonium lactate) dihydroxide (6 mL, 50 wt% in H_2_O) was added to H_2_O (24 mL), and poured into a Teflon-lined stainless-steel autoclave (capacity 50 mL). The solution was heated at 220 °C for 72 h, and cooled in air at ambient temperature. The resulting particles were washed with distilled water and acetone, and dried *in vacuo* to obtain rutile TiO_2_ NRs.

### Au nanoparticle loading

Au NPs were loaded on TiO_2_ by the deposition–precipitation method using urea as the neutralizer.^20^ The TiO_2_ particles were pre-heated at 923 K for 4 h to avoid the influence of the change in the properties of TiO_2_ with heating at various temperatures during the following deposition–precipitation process. TiO_2_ nanoparticles (1 g) were dispersed in an aqueous solution (50 mL) of HAuCl_4_ (4.86 mM) and urea (1.46 g), and stirred at 353 K for 18 h. The resulting particles were washed with hot-distilled water (323 K) 10 times and acetone. After drying *in vacuo*, the particles were calcined by varying the heating temperature (673 K ≤ *T*_c_ ≤ 873 K) and time (1 h ≤ *t*_c_ ≤ 24 h) to obtain Au/TiO_2_.

### Catalyst characterization

For quantifying the loading amount of Au, Au NPs loaded on TiO_2_ were dissolved by using aqua regia, and the concentration was determined by inductively coupled plasma spectroscopy. The samples for TEM and HR-TEM observation were prepared by dropping a suspension of the samples in ethanol onto a copper grid with a carbon support film (grid-pitch 150 μm, Okenshoji Co., Ltd, #10-1006). The measurements were performed by using a JEOL JEM-2100F at an applied voltage of 200 kV. The Au particle size (*d*_Au_) was determined from the image analysis of ∼200 Au particles observed in TEM images. Diffuse reflectance UV-vis-NIR absorption spectra were measured by using a UV-2600 spectrometer (Shimadzu) with an integrating sphere unit (Shimadzu, ISR-2600Plus). BaSO_4_ was used as a reference for the reflectance (*R*_∞_). The Kubelka–Munk function [*F*(*R*_∞_) = (1 − *R*_∞_)^2^/2*R*_∞_] was used for expressing the relative absorption coefficient. The measurement was carried out by using sample powder without dilution. X-ray photoelectron spectra (XPS) were collected by using a Kratos Axis Nova X-ray photoelectron spectrometer at 15 kV and 10 mA using Al Kα as the X-ray source. For the energy reference, the peak of C 1s (284.6 eV) was used.

### Cinnamyl alcohol oxidation

Au/TiO_2_ (5 mg) was dispersed in a solution (20 mL, H_2_O : acetonitrile = 99 : 1 v/v) of cinnamyl alcohol (0.5 mM) in a test tube placed in a double jacket type reaction cell (80 mm in length and 15 mm in diameter). Acetonitrile was used for the complete dissolution of cinnamyl alcohol. After stirring for 0.5 h at 298 K in the dark, visible light was irradiated by means of a 300 W Xe lamp (HX-500, Wacom) with an optical filter Y-51 (*λ*_ex_ > 490 nm, AGC TECHNO GLASS) with stirring for 600 rpm while circulating thermostated water (298 K) through an outer jacket around the cell during the reaction. The light intensity integrated from 420 to 485 nm (*I*_420–485_) was adjusted to 3.3 mW cm^−2^. The cinnamaldehyde yield and selectivity were determined by UV-vis spectroscopy (Shimadzu, UV-1800) and high-performance liquid chromatography (Shimadzu, LC-6 AD, SPD-6 A, C-R8A) [conditions: Shim-pack CLC-ODS (4.6 mm × 150 mm); MeOH–H_2_O (7 : 3 v/v); flow rate = 1.0 mL min^−1^; *λ* = 300 nm]. To confirm the reproducibility of the data, the photocatalytic activity was evaluated by repeating the reaction more than twice for each sample.

### Benzylamine oxidation

Au/TiO_2_ (5 mg) Au/TiO_2_ (5 mg) was dispersed in an acetonitrile solution (20 mL) of benzylamine (0.1 mM) in a test tube placed in a double jacket type reaction cell (80 mm in length and 15 mm in diameter). After stirring for 0.5 h at 298 K in the dark, visible light was irradiated by means of a 300 W Xe lamp (HX-500, Wacom) with an optical filter Y-45 (*λ*_ex_ > 430 nm, AGC TECHNO GLASS) with stirring for 600 rpm while circulating thermostated water (298 K) through an outer jacket around the cell during the reaction. The light intensity (*I*_420–485_) was adjusted to 6.0 mW cm^−2^. The benzaldehyde yield and selectivity were determined by UV-vis spectroscopy (Shimadzu, UV-1800) and high-performance liquid chromatography (Shimadzu, LC-6 AD, SPD-6 A, C-R8A) [conditions: Shim-pack CLC-ODS (4.6 mm × 150 mm); acetonitrile; flow rate = 0.5 mL min^−1^; *λ* = 280 nm]. To confirm the reproducibility of the data, the photocatalytic activity was evaluated by repeating the reaction more than twice for each sample.

### Decomposition of 2-naphthol

Au/TiO_2_ (5 mg) was dispersed in a solution (20 mL, H_2_O : acetonitrile = 99 : 1 v/v) of 2-naphthol (10 μM) in a test tube placed in a double jacket type reaction cell (80 mm in length and 15 mm in diameter). Acetonitrile was used for the complete dissolution of 2-naphthol. After stirring for 0.25 h at 298 K in the dark, visible light was irradiated by means of a 300 W Xe lamp (HX-500, Wacom) with an optical filter Y-45 (*λ*_ex_ > 430 nm, AGC TECHNO GLASS) with stirring for 600 rpm while circulating thermostated water (298 K) through an outer jacket around the cell during the reaction. The light intensity (*I*_420–485_) was adjusted to 6.0 mW cm^−2^. The concentration of 2-naphthol was quantified by high-performance liquid chromatography (Shimadzu, LC-6 AD, SPD-6 A, C-R8A) [conditions: Shim-pack CLC-ODS (4.6 mm × 150 mm); MeOH–H_2_O (7 : 3 v/v); flow rate = 1.0 mL min^−1^; *λ* = 223 nm]. To confirm the reproducibility of the data, the photocatalytic activity was evaluated by repeating the reaction more than twice for each sample.

### Oxygen evolution reaction

Au/TiO_2_ (10 mg) and La_2_O_3_ (20 mg) was dispersed in an aqueous solution (10 mL) of AgNO_3_ (10 mM) in a test tube placed in a double jacket type reaction cell (80 mm in length and 15 mm in diameter). La_2_O_3_ was used for maintaining a constant pH (pH 7.45).^21^ After removing the dissolved O_2_ by Ar bubbling for 0.5 h, visible light was irradiated by using a xenon lamp with an optical filter Y-49 (*λ*_ex_ > 470 nm, AGC TECHNO GLASS) with stirring for 600 rpm while circulating thermostated water (298 K) through an outer jacket around the cell during the reaction. The light intensity integrated from 470–780 nm was adjusted to 30 mW cm^−2^. The amount of O_2_ evolved was measured by gas chromatography (Shimadzu. GC-8APT) with a thermal conductivity detector [conditions: a molecular sieve 5A column (*ϕ* 3.0 mm × 1 m), carrier gas = Ar]. The injection and column temperatures were set at 60 °C. To confirm the reproducibility of the data, the photocatalytic activity was evaluated by repeating the reaction more than twice for each sample.

### Finite difference time domain (FDTD) simulations

Au/anatase TiO_2_ and Au/rutile TiO_2_ were modelled with an Au hemisphere (HS Au) and an Au truncated octahedron (t-Oh Au) loaded on a TiO_2_ slab (650 × 650 × 50 nm^3^), respectively. Particle sizes obtained from the TEM observations were used as the size of the modelled Au particles. The optical response of the models was reproduced on the basis of complex refractive indices for each constituent from the literature.^22^ Light scattering behavior of the models was simulated using a total-field scattered-field (TFSF) source. An *x*-polarized plane wave with a wavelength from 300 to 1200 nm (*f* = 250 – 1000 THz) was irradiated on the top of the Au particle from the *z*-axis direction perpendicular to the TiO_2_ slab. Local electric field images were obtained by using *E*-filed monitors located on the *xy* plane (*z* = 0) and the *xz* plane (*y* = 0). Considering the balance between the calculation cost and accuracy, mesh refinement and a perfectly matched layer (PML) with symmetric and/or anti-symmetric boundary conditions were imposed on the models. All of the FDTD simulations were carried out with an Ansys Lumerical FDTD simulation program package.

## Results and discussion

### Preparation and characterization of Au/TiO_2_

**Fig. 1:**
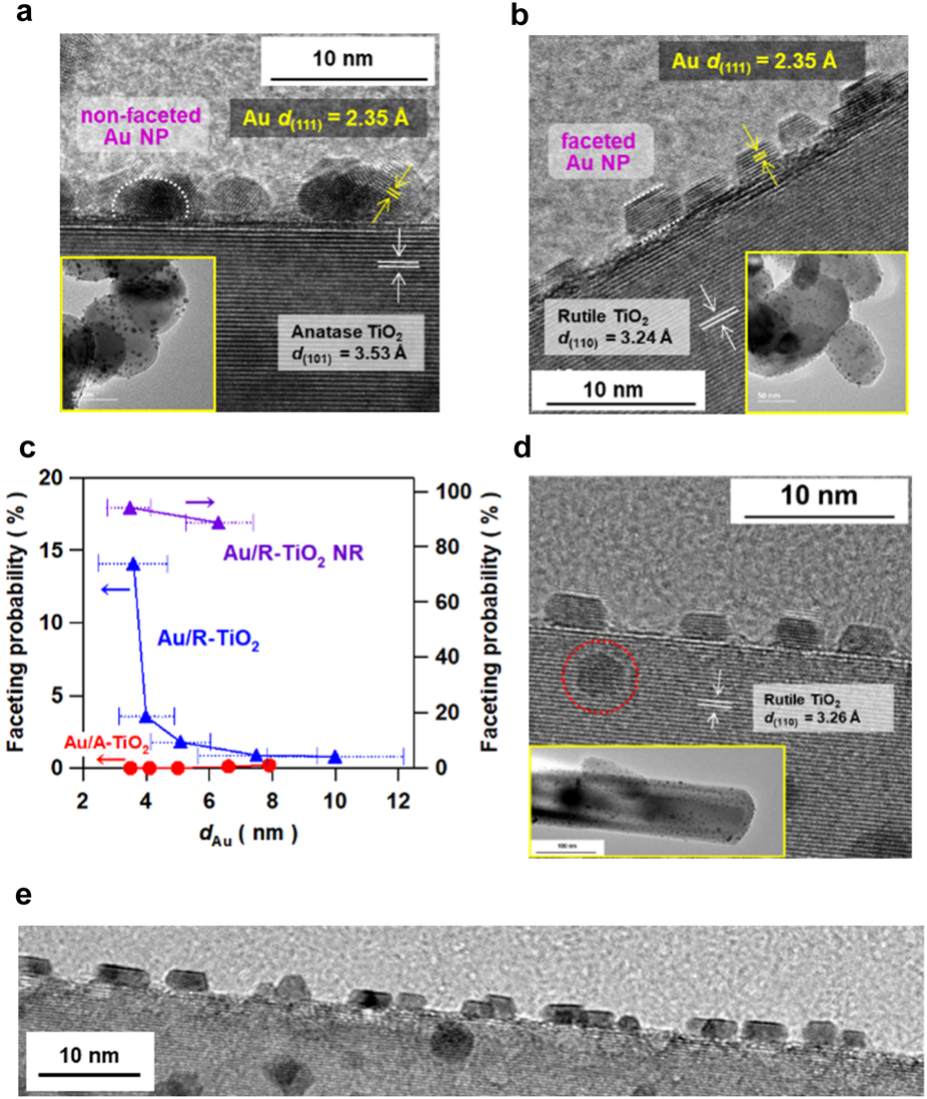
HR-TEM images of Au/anatase TiO_2_ (Au/A-TiO_2_) (a) and Au/rutile TiO_2_ (Au/R-TiO_2_) (b) prepared at heating temperature *T*_c_ = 673 K and time *t*_c_ = 1 h. The insets show the TEM images of the samples. (c) Faceting probability of Au NPs on A-TiO_2_ (red), R-TiO_2_ (blue), and rutile TiO_2_ nanorod (R-TiO_2_ NR, violet) particles as a function of *d*_Au_. The length of the bars for *d*_Au_ expresses the standard deviation of the Au particle size for each sample. (d) HR-TEM image of Au/R-TiO_2_–NR prepared at *T*_c_ = 673 K and *t*_c_ = 1 h. The inset is the TEM image of the sample. (e) HR-TEM image of Au/R-TiO_2_–NR prepared at *T*_c_ = 673 K and *t*_c_ = 1 h in a wide field of view.

Au NPs were loaded on irregular-shaped commercial A-TiO_2_ particles (mean particle size = 150 nm and specific surface area = 8.1 m^2^ g^−1^) and R-TiO_2_ particles (mean particle size = 80 nm and specific surface area = 18 m^2^ g^−1^) by the deposition precipitation method.^20^ Prior to the deposition of Au NPs, cleanness of the TiO_2_ surface was confirmed by X-ray photoelectron (XP) spectroscopy (Fig. S1†). The loading amount of Au (*x*_Au_) was quantified to be 4.22 ± 0.03 mass% for A-TiO_2_ and 4.25 ± 0.07 mass% for R-TiO_2_ by inductively coupled plasma spectroscopy (Table S2†). In the high resolution-transmission electron microscopy (HR-TEM) images of the samples prepared at heating temperature (*T*_c_) = 673 K and heating time (*t*_c_) = 1 h (Fig. 1a, b and S2†), the *d*-spacings of the supports are in agreement with the values of A-TiO_2_(101) (3.53 Å) and R-TiO_2_(110) (3.24 Å), respectively. Also, the *d*-spacing of the deposits in each sample is equal to the value of Au(111) (2.35 Å). Au NPs with mean sizes (*d*_Au_) of 3.5 nm and 3.6 nm are highly dispersed on A-TiO_2_ and R-TiO_2_ particles, respectively. Intriguingly, faceted Au NPs with sharp edges and corners are observed on R-TiO_2_ particles, while every Au NP on A-TiO_2_ is hemisphere-like (HS).

The size of Au NPs was controlled with the loading amount maintained constant by changing *T*_c_ and *t*_c_ during the deposition precipitation process (Fig. S3–S5†).^23^ The *d*_Au_ varies from 3.5 nm to 7.9 nm in the Au/A-TiO_2_ system, and from 3.6 nm to 10.0 nm in the Au/R-TiO_2_ system with increasing heating temperature (673 K ≤ *T*_c_ ≤ 873 K) and time (1 h ≤ *t*_c_ ≤ 24 h). Faceting probability (*P*_f_) of Au/TiO_2_ was defined as the ratio of the number of faceted Au NPs to the total number of Au NPs. In this study, hemispherical Au NPs (Fig. 1a, white dotted semicircle) and Au NPs with a sharp truncated top plane parallel to the interface with TiO_2_ (Fig. 1b, white dotted parallel lines) are assigned to the non-faceted and faceted particles, respectively. The basis for these criteria is described in the next section. To minimize the arbitrariness, the *P*_f_ was calculated by counting the numbers of faceted and non-faceted Au NPs on the edge of TiO_2_ particles in the TEM images over 10^3^ particles (Fig. S6, Table S2†). While the *P*_f_ is negligibly small in the Au/A-TiO_2_ system (<0.2%) regardless of *d*_Au_, the *P*_f_ increases with a decrease in *d*_Au_ from 0.8% at *d*_Au_ = 10.0 nm to 14% at *d*_Au_ = 3.6 nm in the Au/R-TiO_2_ system (Fig. 1c). Although NPs can appear non-faceted if imaged off-axis as TEM shows a projection on the NPs, the very small *P*_f_ for the Au/A-TiO_2_ system is not for this. Actually, not faceted but hemispherical Au NPs are usually observed in many TEM images of Au/A-TiO_2_ reported so far.

### Interface analysis of Au/TiO_2_

**Fig. 2:**
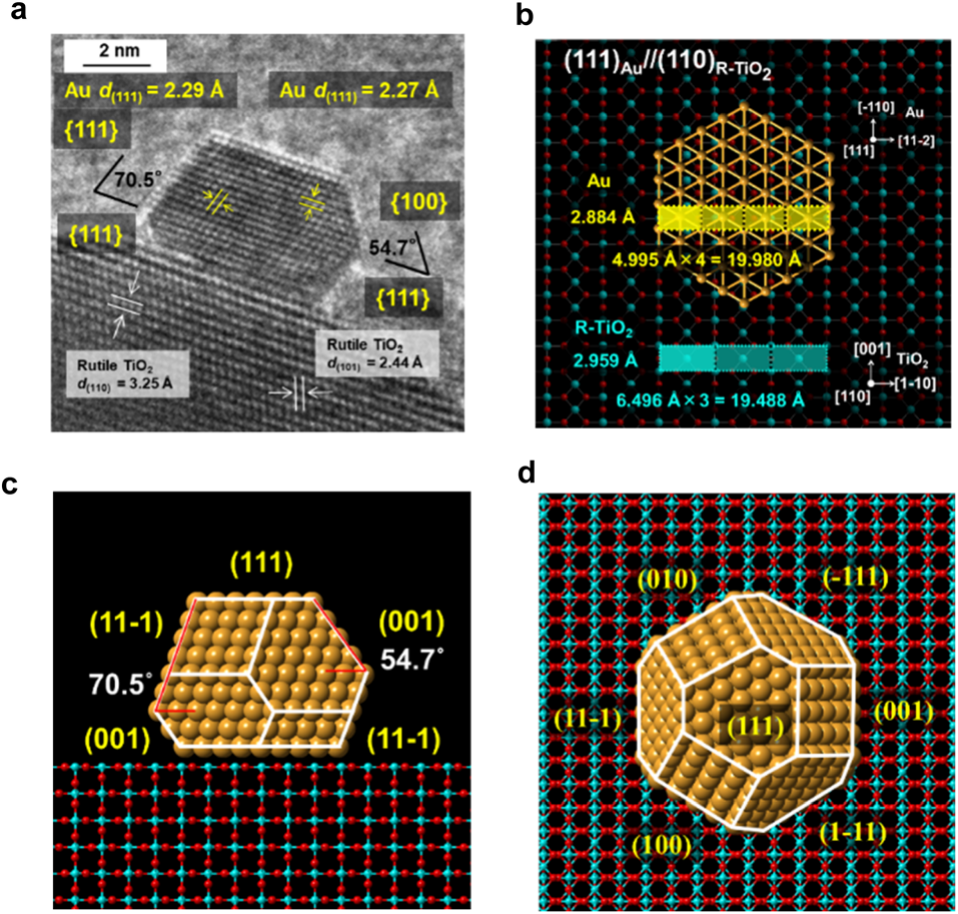
(a) HR-TEM image of Au/R-TiO_2_. The heteroepitaxial junction model with an orientation of (111)_Au_//(110)_R-TiO_2__. (b) The domain-matching epitaxy model. The side view (c) and top view (d) of the most thermodynamically stable shape of Au NPs on rutile TiO_2_ constructed by using the Wulff theorem.

The interface between Au NPs and R-TiO_2_ was analyzed by HR-TEM. The HR-TEM image of a typical faceted Au NP on R-TiO_2_ (Fig. 2a) shows that an atomically commensurate junction is formed with an orientation of (111)_Au_//(110)_R-TiO_2__. The two-dimensional rectangular lattices of Au(111) and R-TiO_2_(110) possess the unit dimensions of 4.995 Å × 2.884 Å and 6.496 Å × 2.959 Å, respectively (Fig. 2b). The quadruple and triple of the unit cells almost match with a mismatch of +2.5% and −2.5%, which strongly suggests the formation of a domain-matching epitaxial junction. A previous study by means of electron backscattered diffraction showed the same orientation for an evaporated Au film on the (110) surface of a R-TiO_2_ single crystal followed by annealing at 775 K.^24^ The most thermodynamically stable shape of Au NPs on the R-TiO_2_(110) surface was constructed by using the Wulff theorem using the values for the surface energies of 0.72 J m^−2^ for Au(111) (ref. 25) and 0.40 J m^−2^ for R-TiO_2_(110),^26^ and an interfacial energy of 0.86 J m^−2^ for Au(111) and R-TiO_2_(110).^27^ As shown in the side view and top view (Fig. 2c and d), the Au NP takes the most stable truncated octahedral shape (t-Oh Au NP), and the side view (Fig. 2c) is very similar to that observed in the HR-TEM image (Fig. 2a). Thus, the faceting of Au NPs on R-TiO_2_ can be induced by the crystallographic metal-support interaction between Au(111) and R-TiO_2_(110).

### Plasmonic photocatalytic activity of Au/TiO_2_

**Fig. 3:**
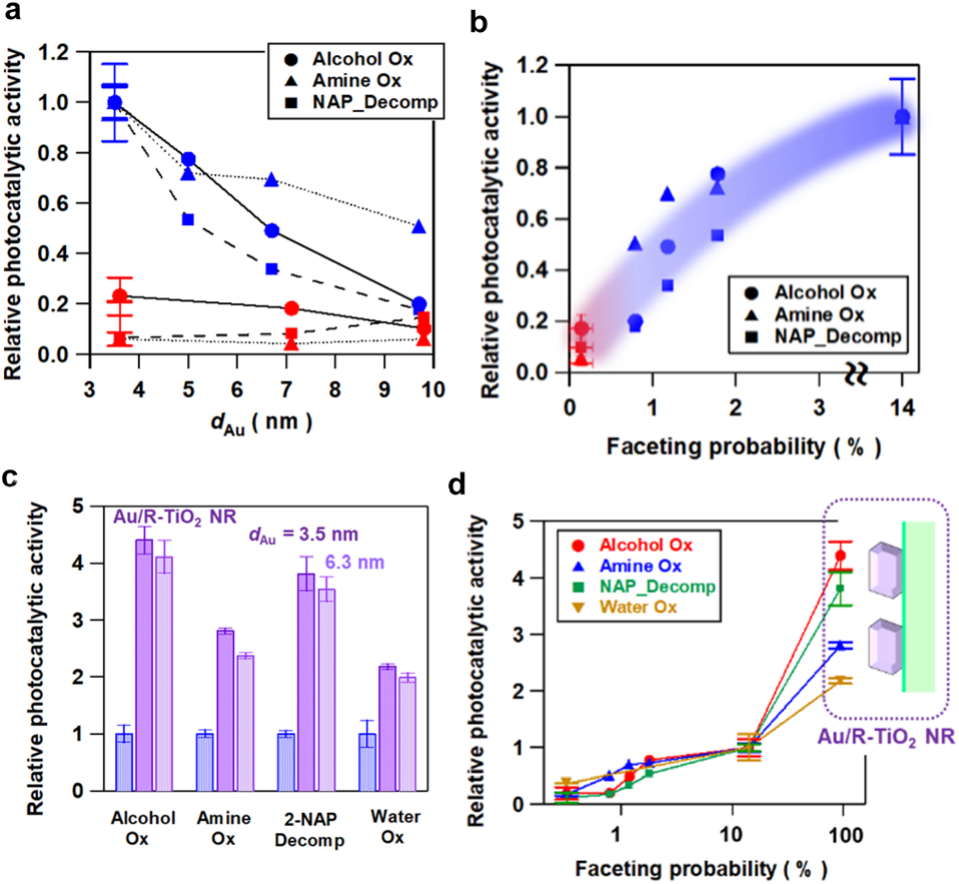
(a) The *d*_Au_ dependence of the relative plasmonic photocatalytic activities of Au/A-TiO_2_ and Au/R-TiO_2_ for the oxidations of cinnamyl alcohol to cinnamaldehyde (Alcohol Ox) and benzylamine to benzaldehyde (Amine Ox), and the oxidative degradation of 2-naphthol (NAP_Decomp). The photocatalytic activity is normalized by using the value of Au/R-TiO_2_ with a *P*_f_ of 14.1% or *d*_Au_ = 3.6 nm. (b) Relation between the photocatalytic activity ratio of Au/TiO_2_ and the *P*_f_ value. (c) Comparison between the photocatalytic activity of Au/R-TiO_2_ (*d*_Au_ = 3.6 nm, *x*_Au_ = 4.2 mass%, *P*_f_ = 14.1%), Au/R-TiO_2_ NR (*d*_Au_ = 3.5 nm, *x*_Au_ = 4.2 mass%, *P*_f_ = 94%) and Au/R-TiO_2_ NR (*d*_Au_ = 6.3 nm, *x*_Au_ = 4.2 mass%, *P*_f_ = 89%). (d) Relation between the photocatalytic activity of Au/TiO_2_ and the *P*_f_ value including the data for Au/R-TiO_2_ NRs with a *P*_f_ of 94%. The photocatalytic activity was evaluated by repeating the reaction more than twice for each catalyst under the same conditions. In all figures, the length in the ordinate shows the scattering of the data.

The Au NP-faceting effect of Au/A-TiO_2_ (red) and Au/R-TiO_2_ (blue) plasmonic photocatalysts on the activities was studied for the oxidation of cinnamyl alcohol to cinnamaldehyde (circles)^28^ and of benzylamine to benzaldehyde (triangles),^29^ and the oxidative degradation of 2-naphthol (squares)^30^ under visible-light irradiation. The action spectra previously confirmed that these reactions proceed *via* the LSPR excitation-driven HET mechanism.^28–30^ Each reaction apparently follows a zero-order rate equation (Fig. S7–S9†), and from the slope in the time course for the reaction, the reaction rate was calculated. Furthermore, the photocatalytic activity is expressed by using the relative reaction rate with respect to the value for Au/R-TiO_2_ with *d*_Au_ = 3.6 nm and *P*_f_ = 14%. The *d*_Au_-dependence of the relative photocatalytic activity for each reaction (Fig. 3a) shows that Au/R-TiO_2_ (blue) possesses photocatalytic activity much higher than that of Au/A-TiO_2_ (red). The photocatalytic activity of Au/R-TiO_2_ increases with a decrease in *d*_Au_, while the *d*_Au_-dependence of the activity is weak in the Au/A-TiO_2_ system. Plots of the photocatalytic activity of Au/A-TiO_2_ (red) and Au/R-TiO_2_ (blue) against *P*_f_ show a clear positive correlation in every reaction system (Fig. 3b). In addition, visible-light irradiation (*λ*_ex_ > 470 nm) of Au/TiO_2_ yields O_2_, the amount of which increases in proportion to irradiation time. The O_2_-generation rate for Au/R-TiO_2_ is 2.7 times higher as compared with the value for Au/A-TiO_2_ (Fig. S10†).^18^

As demonstrated by the interface analysis, the faceting of Au NPs on R-TiO_2_ can be induced by using the heteroepitaxial junction with the orientation of (111)_Au_//(110)_R-TiO_2__. For the equilibrium form of TiO_2_ crystals, the percentage of the R-TiO_2_(110) facet was estimated to be 83% by using the Wulff construction.^31^ A domain matching epitaxial junction can also be formed between Au and A-TiO_2_ with an orientation of (111)_Au_//(001)_A-TiO_2__.^32^ However, the percentage for the A-TiO_2_(001) facet predicted by the Wulff theorem is below 6%.^33^ These considerations qualitatively rationalize the large gap in the *P*_f_ between Au/A-TiO_2_ and Au/R-TiO_2_ (Fig. 1c). However, the *P*_f_ of Au/R-TiO_2_ is much smaller than the percentage of the R-TiO_2_(110) facet thermodynamically predicted. Actually, the area of the (110) domain exposed on the surface limits the *P*_f_ in the ordinary irregular shaped R-TiO_2_ particles, which would further be responsible for the decrease in the *P*_f_ with increasing *d*_Au_. Then, R-TiO_2_ nanorods (NR) with large-area (110) side walls (mean long axis = 0.84 ± 0.22 μm, mean short axis = 87 ± 32 nm, and specific surface area = 6.0 m^2^ g^−1^) were synthesized by a hydrothermal method (Fig. S11†) and used as the support of Au NPs (Au/R-TiO_2_ NRs). Surprisingly, the *P*_f_ at *d*_Au_ = 3.5 nm on the TiO_2_ NRs increases to 94% close to the percentage of the {110} facet area to the total surface area for a rectangular with the R-TiO_2_ NR dimension (95%) (Fig. 1c and Table S2†). The top view of most Au NPs (dotted red circles in Fig. 1d and S12†) has a hexagonal shape that closely resembles that of t-Oh Au NP (Fig. 2d). Furthermore, the photocatalytic activity of Au/R-TiO_2_ NR for the reactions was evaluated under the same conditions. For every reaction, Au/R-TiO_2_ NRs exhibit photocatalytic activity higher than even that of Au/R-TiO_2_ with a *P*_f_ of 14% by a factor of 2.2–4.4, depending on the type of reaction (Fig. 3cand d). Also, in the Au/R-TiO_2_ NR system, the *P*_f_ value only decreases to 89% when the *d*_Au_ increases from 3.5 nm to 6.3 nm (Fig. 1c). The photocatalytic activity of Au/R-TiO_2_ NRs with *d*_Au_ = 6.3 nm and *P*_f_ = 89% for each reaction is slightly lower than that of Au/R-TiO_2_ NRs with *d*_Au_ = 3.5 nm and *P*_f_ = 94% (Fig. 3c). TEM observation for Au/R-TiO_2_ NRs after each reaction confirmed that the structure is almost maintained (Fig. S13†).

The results of the activities of the Au/TiO_2_ plasmonic photocatalysts can be summarized as follows. The Au loading amount of all Au/TiO_2_ samples used in this study is controlled to be almost constant (∼4.2 mass%) (Table S2†). In the Au/A-TiO_2_ system, the *P*_f_ is below 0.2% regardless of the Au particle size (Fig. 1c), and the photocatalytic activity remains low (Fig. 3a). In the Au/R-TiO_2_ system, the photocatalytic activity increases with an increase in the *P*_f_ value (Fig. 3band d). Furthermore, Au/R-TiO_2_ NRs with *d*_Au_ = 3.5 nm and *P*_f_ = 94% shows much higher photocatalytic activity than Au/R-TiO_2_ with *d*_Au_ = 3.6 nm and *P*_f_ = 14% (Fig. 3d), while the photocatalytic activity of Au/R-TiO_2_ NRs with *d*_Au_ = 3.5 nm and *P*_f_ = 94% is comparable with that of Au/R-TiO_2_ NRs with *d*_Au_ = 6.3 nm and *P*_f_ = 89% (Fig. 3c). Au/R-TiO_2_ NRs were confirmed to be stable during each photocatalytic reaction. In the Au/TiO_2_ system, a positive correlation is recognized between the photocatalytic activity and the *P*_f_ value although the degree of the dependence depends on the type of reaction (Fig. 3d). Evidently, the photocatalytic activity of Au/TiO_2_ can be unified to the *P*_f_ value regardless of the TiO_2_ crystal form and Au particle size.

### Physical properties of Au/TiO_2_

XP spectra were measured for Au/TiO_2_ particles, TiO_2_ particles and a Au film-coated glass plate for comparison (Fig. S14 and Table S3†). In the XPS spectra of the Au film/glass, two signals due to the emission from the 4f_7/2_ and 4f_5/2_ orbitals are observed at the binding energy (*E*_B_) = 84.0 eV and 87.7 eV, respectively, close to the literature values of 83.9 eV and 87.6 eV for bulk Au.^34^ The *E*_B_ values of the Au 4f_7/2_ and 4f_5/2_ orbitals for Au/A-TiO_2_ (83.2 ± 0.2 eV and 86.9 ± 0.2 eV) and Au/R-TiO_2_ (83.2 ± 0.1 eV and 86.9 ± 0.2 eV) are significantly smaller than the values for the Au film-coated glass (Fig. S14a, b and Table S3†). On the other hand, Au/A-TiO_2_ and Au/R-TiO_2_ have the Ti 2p_3/2_-*E*_B_ values of 458.6 ± 0.1 eV and 458.5 ± 0.1 eV, respectively, which are slightly larger than the value of 458.3 eV for unmodified R-TiO_2_ (Figure S14c, d and Table S3†). These results suggest the formation of a Schottky junction in the Au/A-TiO_2_ and Au/R-TiO_2_ systems with interfacial electron transfer from TiO_2_ to Au NPs.^12^

**Fig. 4:**
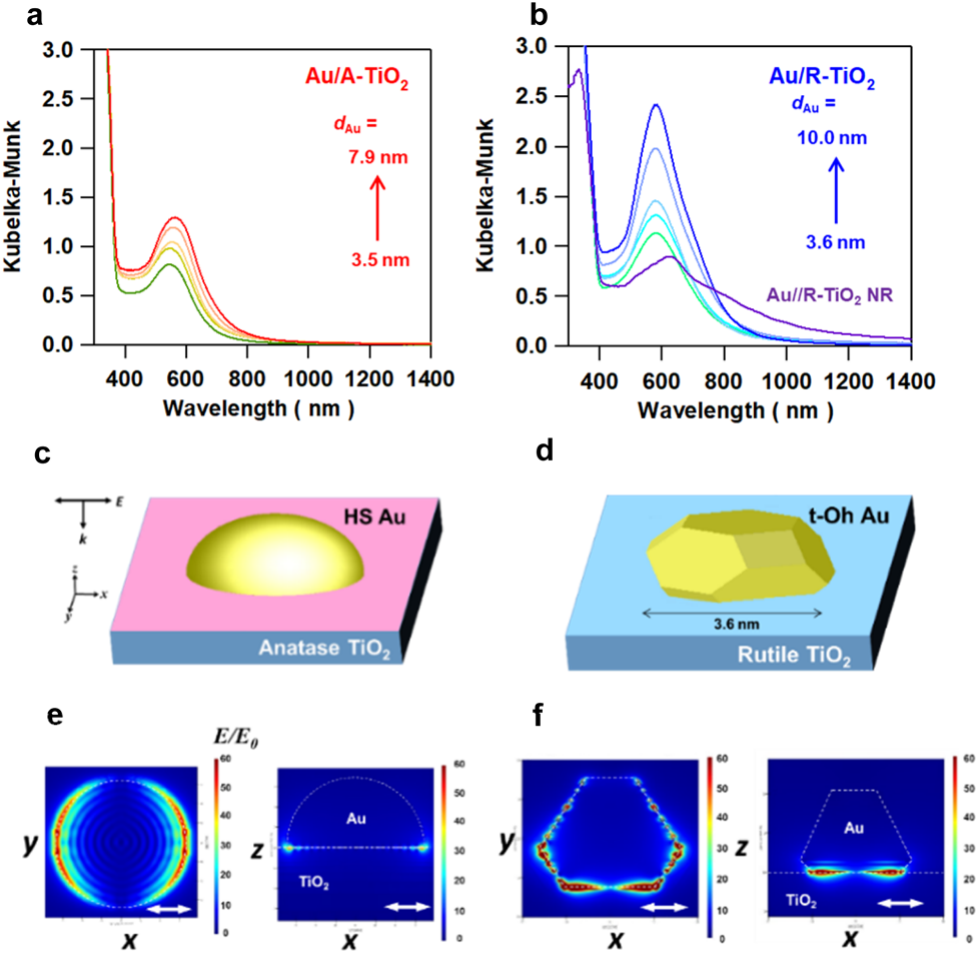
Kubelka–Munk-transformed absorption spectra of Au/A-TiO_2_ (a) and Au/R-TiO_2_ (b) with varying Au particle sizes (*d*_Au_) with Au(*d*_Au_ = 6.3 nm)/R-TiO_2_ NRs. FDTD-simulation models for HS Au(*d*_Au_ = 3.5 nm)/A-TiO_2_ (c) and t-Oh Au(*d*_Au_ = 3.6 nm)/R-TiO_2_ (d). 3D-FDTD-calculated local electric field distribution for HS Au(*d*_Au_ = 3.5 nm)/A-TiO_2_ (e) and t-Oh Au(*d*_Au_ = 3.6 nm)/R-TiO_2_ (f) excited with *x*-polarized light incident from the *z*-axis direction (*k*//*z*, *E*//*x*) at wavelengths of 702 and 704 nm, respectively. The Au particle-TiO_2_ slab interface is set to the origin of the *z*-axis. The white double-headed arrows represent the polarization direction of the injected light.

Optical properties of Au/A-TiO_2_ and Au/R-TiO_2_ were studied by diffuse reflectance UV-visible-near infrared spectroscopy. In each Kubelka–Munk-transformed absorption spectrum of Au/A-TiO_2_ (Fig. 4a) and Au/R-TiO_2_ (Fig. 4b), the LSPR peak of Au NPs is observed in the 550–600 nm range with absorption due to the Au interband transition from the Au 5d band to 6sp at < ∼600 nm.^35^ The LSPR significantly intensifies with the peak redshifted with increasing the Au particle size from 551 nm at *d*_Au_ = 3.5 nm to 572 nm at *d*_Au_ = 7.9 nm in the Au/A-TiO_2_ system and from 583 nm at *d*_Au_ = 3.6 nm to 589 nm at *d*_Au_ = 10.0 nm in the Au/R-TiO_2_ system. Uniquely, in the Au/R-TiO_2_ NR system, the main LSPR peak redshifts to 620 nm with two shoulders at around 550 nm and 800 nm. Furthermore, the spatial distribution of the local electric field was calculated by the 3D-FDTD method for the models of Au(*d*_Au_ = 3.5 nm)/A-TiO_2_ (Fig. 4c) and Au(*d*_Au_ = 3.6 nm)/R-TiO_2_ (Fig. 4d) upon excitation by light with wavelength (*λ*_ex_) = 702 nm and 704 nm, respectively. In the former, the local electric field is fairly uniformly distributed along the perimeter of the Au NPs at the interface with A-TiO_2_ (Fig. 4e). In contrast, the LSPR excitation of the latter generates a highly concentrated electric field or “plasmonic hot spots” at the edges and corners of the faceted Au NPs near the interface with R-TiO_2_ (Fig. 4f). The enhancement factor (EF) defined by the square of the ratio of the local electric field intensity to the electric field intensity of incident light (|*E*|^2^/|*E*_0_|^2^) was calculated (Table S4†). The excited localized electromagnetic fields were monitored at the *xz* and *xy* planes of the models. Remarkably, the maximum EF in the t-Oh Au/R-TiO_2_ system reaches 7.4 × 10^4^ at the *xz* plane and 2.1 × 10^5^ at the *xy* plane, which are greater than the values of 2.5 × 10^3^ at the *xz* plane and 3.6 × 10^3^ at the *xy* plane in the HS-Au/A-TiO_2_ system by factors of 30 and 58, respectively. Clearly, the local electric field generated by the LSPR excitation is drastically enhanced by the heteroepitaxial junction-induced faceting of Au NPs on R-TiO_2_.

## Discussion

The overall efficiency in the HET-type plasmonic photocatalytic reactions (*ϕ*_all_) can be expressed by multiplication of the efficiencies of a series of physical events including light harvesting (LHE), hot-electron injection (*ϕ*_HEI_), and charge separation (*ϕ*_CS_), and the efficiency of the surface chemical reaction (*ϕ*_chem_). Among the factors, the strength of the local electric field induced by LSPR excitation (*E*) is decisively important because the overall efficiency is proportional to the square of |*E*|(eqn (1)).^36,37^

1 *ϕ*_all_ = *ϕ*_phys_ × *ϕ*_chem_ ∝ (*A*/*γ*_rel_)|*E*|^2^*ϕ*_chem_

where *ϕ*_phys_ = LHE × *ϕ*_HEI_ × *ϕ*_CS_, *A* is the area of the Au NP/semiconductor interface, and *γ*_rel_ is the energy relaxation rate of the electron gas.

The LSPR excitation of Au/R-TiO_2_ induces “plasmonic hot spots”, in which the local electric field is much more intense than that of the Au/A-TiO_2_ system, at the edges and corners of t-Oh Au NPs around the perimeter interface with TiO_2_. As a result, light harvesting or the rate of hot carrier generation in Au NPs is enhanced near the interface (LHE ↑). This has recently been experimentally evidenced by means of Kelvin probe force microscopy for Ag NPs on GaN,^38^ and photoconductive atomic force microscopy for the Au nanoprism on TiO_2_ (ref. 39) and GaN.^40^ In this case, it is worth noting that the absorption intensity in the absorption spectrum is not directly related to the photocatalytic activity (Fig.3a, d, 4a and b). The hot electrons are efficiently injected into the CB of TiO_2_ through the large-area (*A* ↑) and high-quality interface (*ϕ*_HEI_ ↑).^41,42^ In the Au/TiO_2_ system, the CB-band bending generated by the Schottky junction can assist charge separation (*ϕ*_CS_ ↑).^12^ In this manner, the finding that the activity of the Au/TiO_2_ plasmonic photocatalysts for various reactions is governed by the *P*_f_ of Au NPs can be explained in terms of the enhancement of the efficiencies in the photophysical processes. In a recent study on the OER by using Au/TiO_2_-nanotube array plasmonic electrodes, the photocurrent has been reported to increase with a decrease in the Au particle size due to the lowering in the Schottky barrier at the Au/TiO_2_ interface.^43^ In the Au/TiO_2_ plasmonic photocatalyst system, this effect seems to be minor because the photocatalytic activities of Au/TiO_2_ are completely different in spite of the comparable Au particle size, *i.e.*, Au(*d*_Au_ = 3.5 nm)/R-TiO_2_ NR ≫ Au(*d*_Au_ = 3.6 nm)/R-TiO_2_ ≫ Au(*d*_Au_ = 3.5 nm)/A-TiO_2_. On the other hand, the faceting effect of Au NPs on the plasmonic photocatalytic activity depends on the type of reaction (Fig. 3d). This fact also points to the importance of the chemical effect or *ϕ*_chem_ for increasing the overall reaction efficiency through optimization according to the reaction.

## Conclusions

This study has provided the following important results on the typical plasmonic photocatalysts of Au/TiO_2_. First, the faceting of Au NPs is induced on R-TiO_2_ by the heteroepitaxial junction with the (111)_Au_//(110)_R-TiO_2__ orientation, while hemispherical Au NPs are exclusively formed on A-TiO_2_. Second, the faceting probability of Au NPs, particularly on the ordinary irregular shaped R-TiO_2_ particle, increases with decreasing Au particle size. Third, a positive correlation is valid between the plasmonic photocatalytic activity of Au/TiO_2_ and the faceting probability of Au NPs on TiO_2_ regardless of the TiO_2_ crystal form and Au particle size. Most importantly, the use of R-TiO_2_ NR with large-area (110) side walls as the support of Au NPs increases the *P*_f_ to over 90%, drastically enhancing the photocatalytic activity. While the technique for the synthesis of various faceted semiconductor NPs is rapidly in progress,^44^ the importance of the intimate junction between metal NPs and semiconductors is being recognized in the field of photocatalysis.^15,45^ This study has shown that the combination of crystal facet engineering and atom-level-interface control can be a powerful and versatile methodology for enhancing the photocatalytic activity of metal–semiconductor nanohybrids for various solar-to-chemical transformations.

## Data availability

Many data have already been shown in ESI.†

## Author contributions

S. N., A. A. and Y. M. conducted catalyst synthesis, characterization, and photocatalytic reactions, M. F. performed FDTD simulations, and H. T. supervised the work and data analysis.

## Conflicts of interest

There are no conflicts to declare.

## Supplementary Material

SC-013-D2SC03549A-s001
