# Supplementary material for: Crystallographic interface control of the plasmonic photocatalyst consisting of gold nanoparticles and titanium(iv) oxide
Source: Chem Sci. 2022 Oct 14;13(42):12340–7. doi: 10.1039/d2sc03549a (PMC9628983; doi:10.1039/d2sc03549a)
Supplement: SC-013-D2SC03549A-s001 [file SC-013-D2SC03549A-s001.pdf]

Electronic Supplementary Material (ESI) for Chemical Science.
This journal is © The Royal Society of Chemistry 2022

Electronic supplementary information

Crystallographic interface control of the plasmonic photocatalyst consisting of gold nanoparticles and titanium(IV) oxide

Shin-ichi Naya,[a] Atsunobu Akita,[b] Yoko Morita,[b] Musashi Fujishima,[b,c] and Hiroaki Tada[b,c]*

[a] Environmental Research Laboratory, Kindai University, 3-4-1, Kowakae, Higashi-Osaka, Osaka 577-8502, Japan.

[b] Graduate School of Science and Engineering, Kindai University, 3-4-1, Kowakae, Higashi-Osaka, Osaka 577-8502, Japan.

[c] Department of Applied Chemistry, Faculty of Science and Engineering, Kindai University, 3-4-1, Kowakae, Higashi-Osaka, Osaka 577-8502, Japan.

* To whom correspondence should be addressed: TEL: +81-6-6721-2332, FAX: +81-6-6727-2024,

E-mail: h-tada@apch.kindai.ac.jp.

Table of contents

1. Supplementary Figures··································S3-11
2. Supplementary Tables··································S12-16

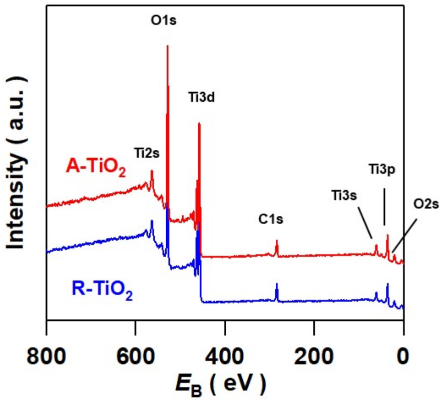

Fig. S1. Wide-scan XP spectra of anatase TiO_2 (A-TiO_2) and rutile TiO_2 (R-TiO_2).

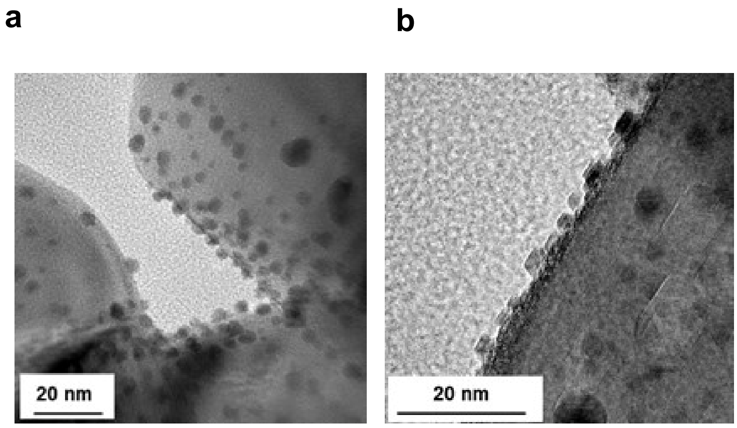

Fig. S2. TEM images of Au/A-TiO_2 (a) and Au/R-TiO_2 (b).

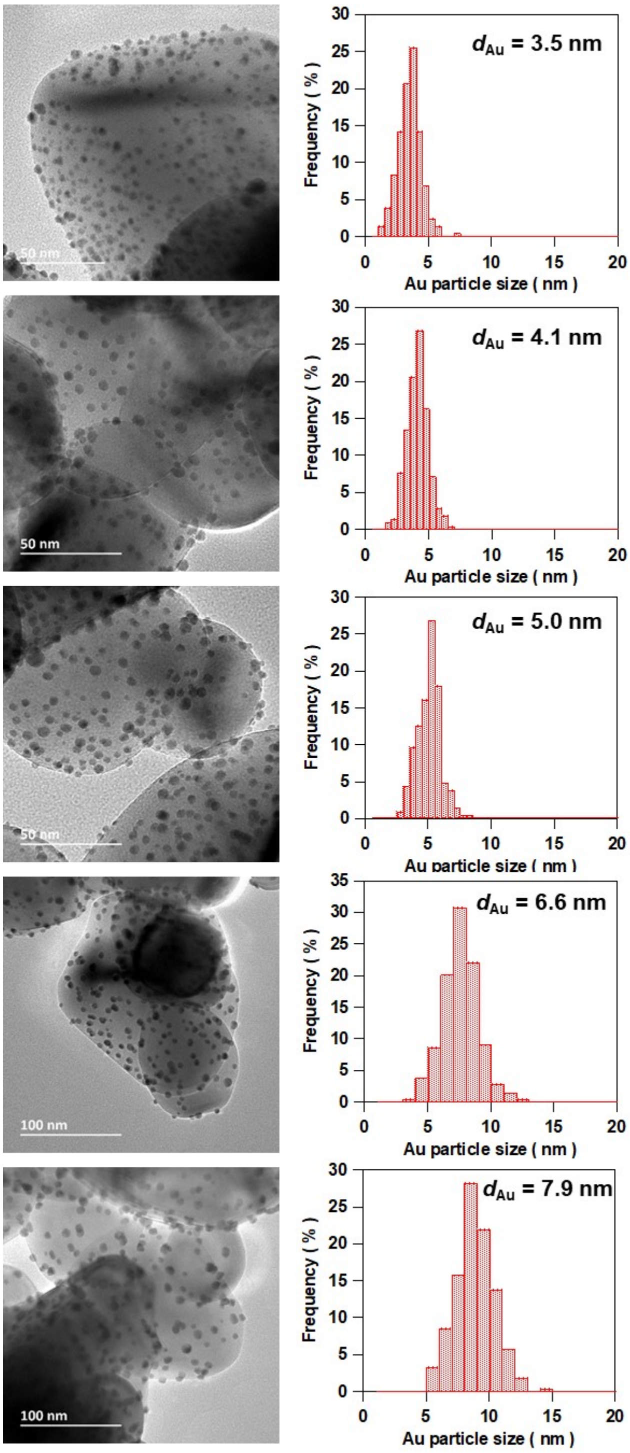

Fig. S3. TEM images and Au size distributions of Au/A-TiO_2 prepared by the deposition precipitation under different conditions.

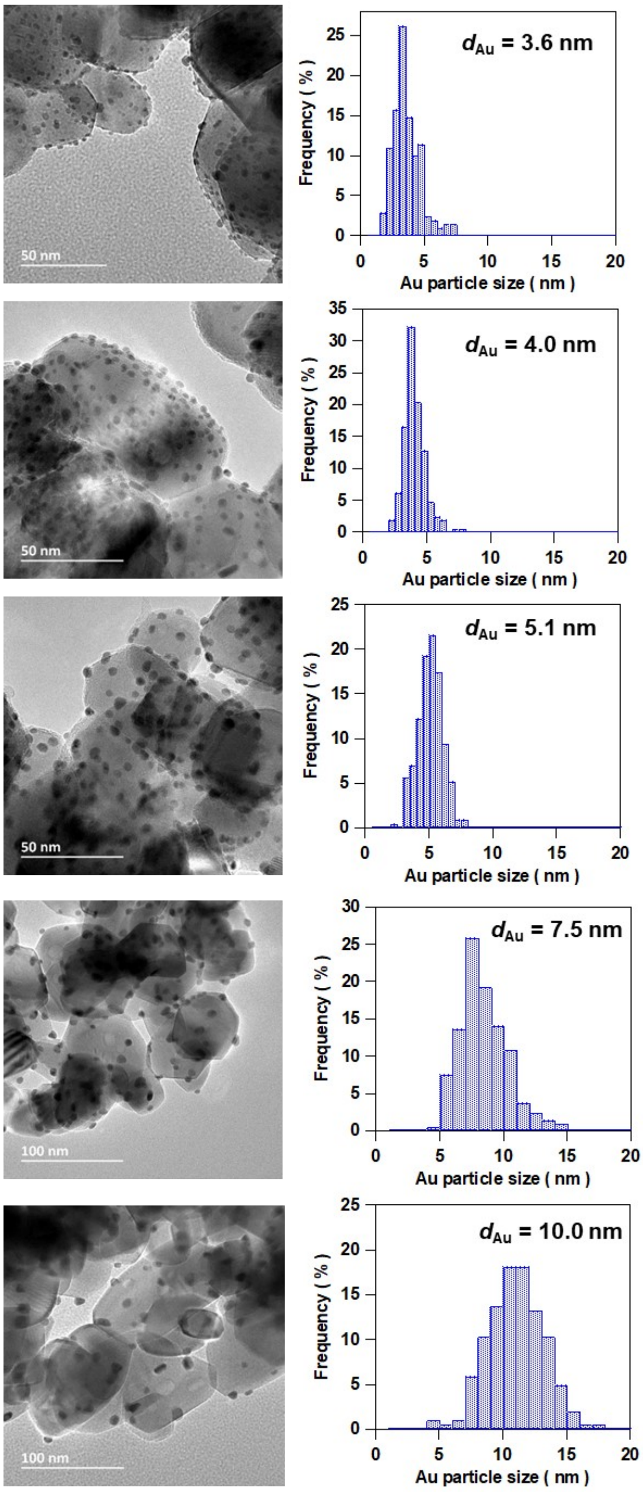

Fig. S4. TEM images and Au size distributions of Au/R-TiO_2 prepared by the deposition precipitation under different conditions.

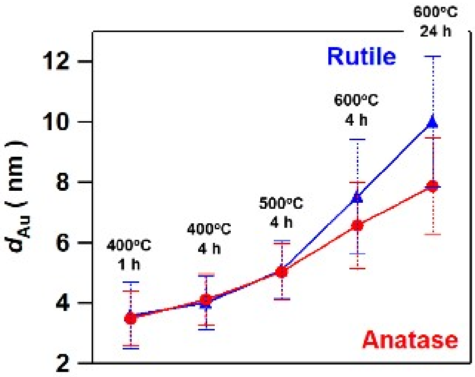

Fig. S5. Au particle size control on anatase and rutile TiO_2 particles. The calcination temperature and time of Au/TiO_2 are shown above each data point.

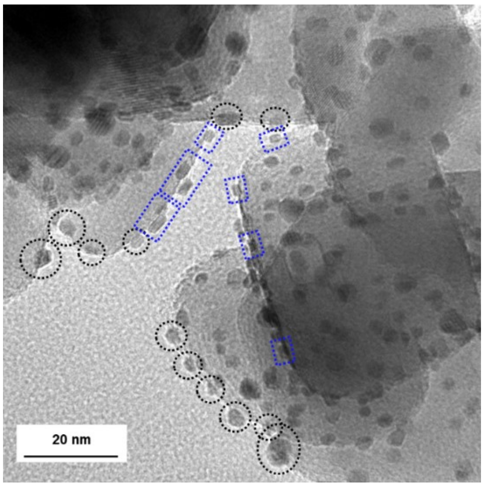

Fig. S6. TEM image of Au/R-TiO_2 prepared at T_c = 673 K and t_c = 1 h. Faceted and non-faceted Au NPs are shown by dotted blue rectangle and black circle.

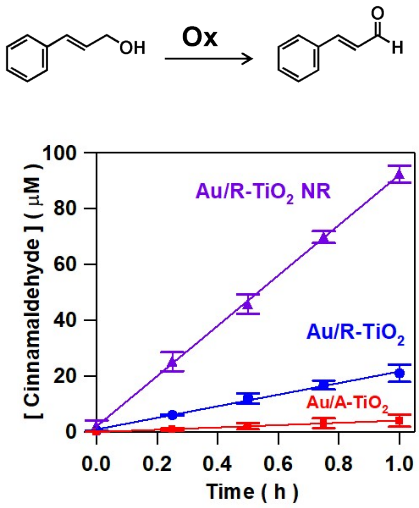

Fig. S7. Time courses for cinnamyl alcohol oxidation under visible-light irradiation (λ_{ex} > 490 nm, light intensity integrated from 420 to 485 nm = 3.3 mW cm^{-2}) in the presence of Au/A-TiO_2 or Au/R-TiO_2 or Au/R-TiO_2 NR.

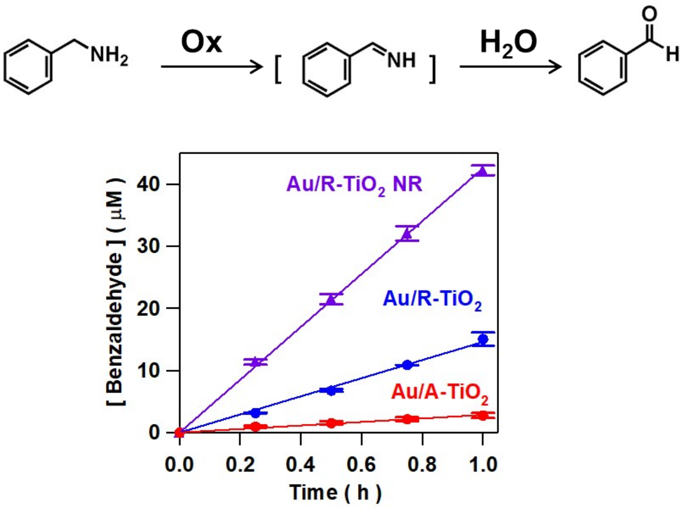

Fig. S8. Time courses for benzylamine oxidation under visible-light irradiation (λ_{ex} > 430 nm, light intensity integrated from 420 to 485 nm = 6.0 mW cm^{-2}) in the presence of Au/A-TiO_2 or Au/R-TiO_2 or Au/R-TiO_2 NR.

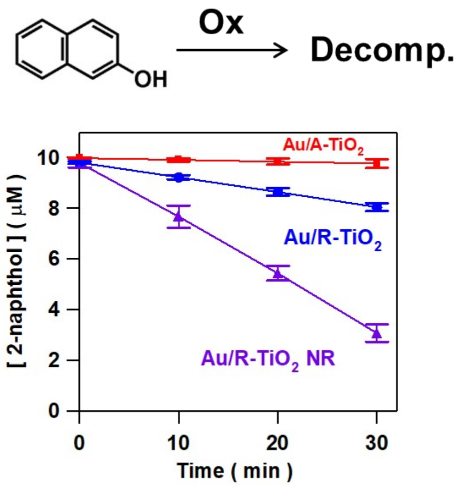

Fig. S9. Time courses for 2-naphthol decomposition under visible-light irradiation (λ_{ex} > 430 nm, light intensity integrated from 420 to 485 nm = 6.0 mW cm^{-2}) in the presence of Au/A-TiO_2 or Au/R-TiO_2 or Au/R-TiO_2 NR.

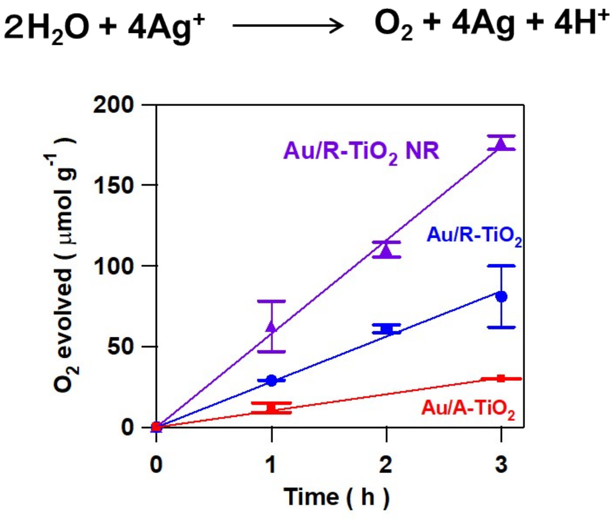

Fig. S10. Time courses for O_2 evolution from 10 mM aqueous solution of $AgNO_3$ (10 mL) containing La_2O_3 (20 mg) under visible-light irradiation (λ_{ex} > 470 nm, light intensity = 30 mW cm^{-2}) in the presence of Au/A-TiO_2 or Au/R-TiO_2 or Au/R-TiO_2 NR (10 mg).

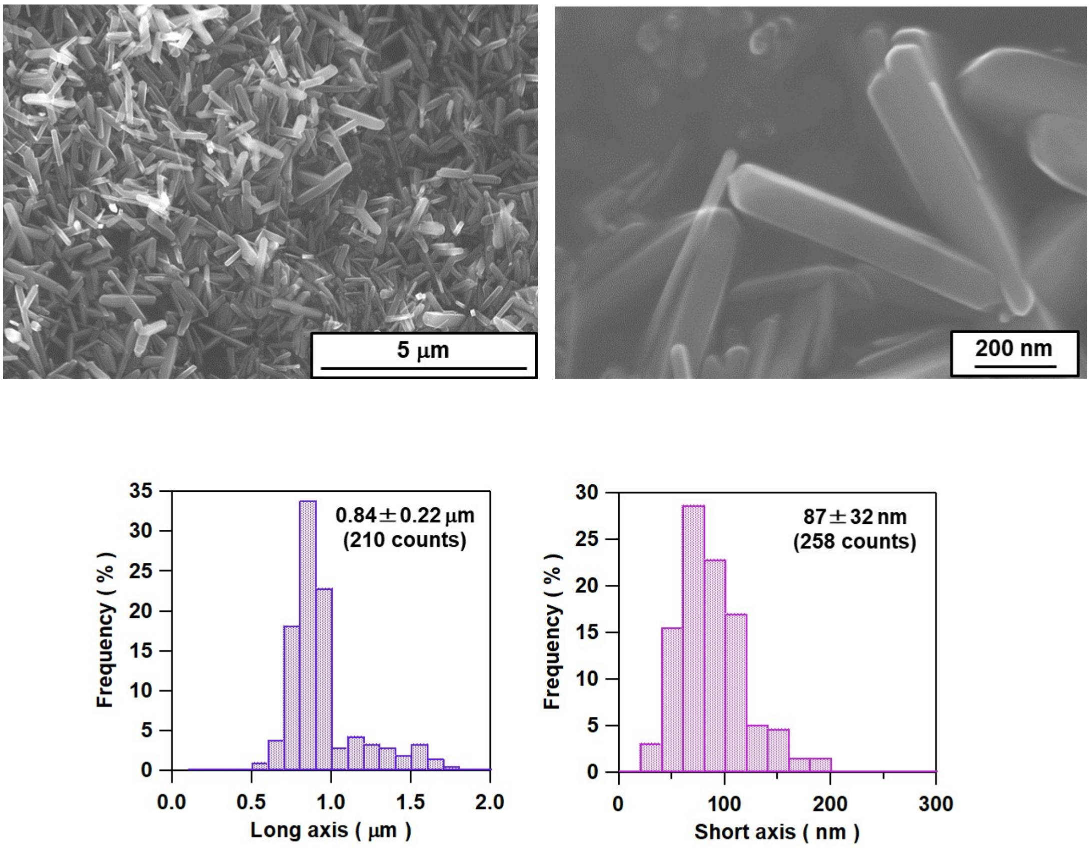

Fig. S11. SEM images and distribution of long and short axis length of R-TiO_2 NR.

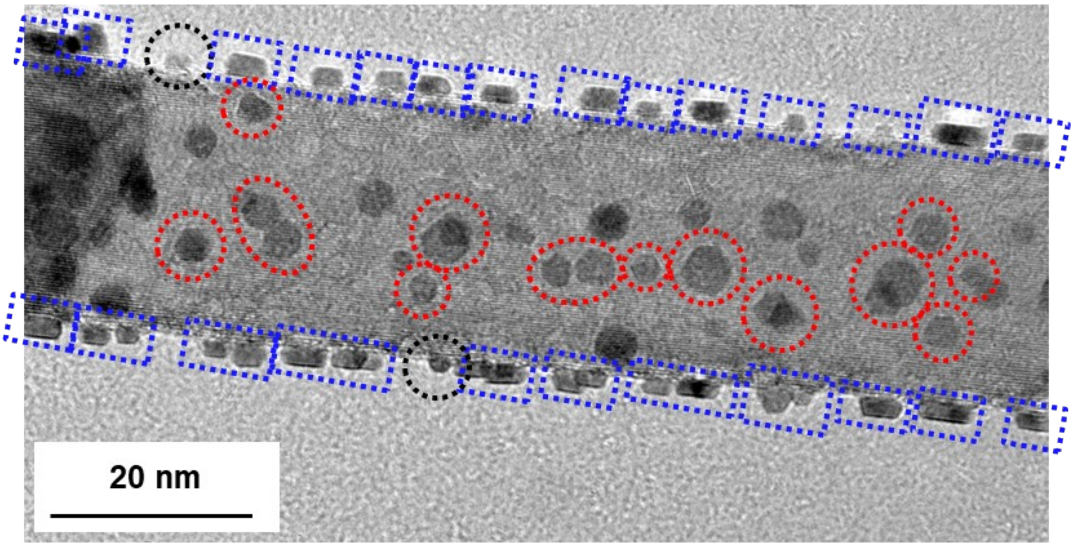

Fig. S12. TEM image of Au/R-TiO_2 NR prepared at T_c = 673 K and t_c = 1 h. Faceted and non-faceted Au NPs are shown by dotted blue rectangle and black circle.

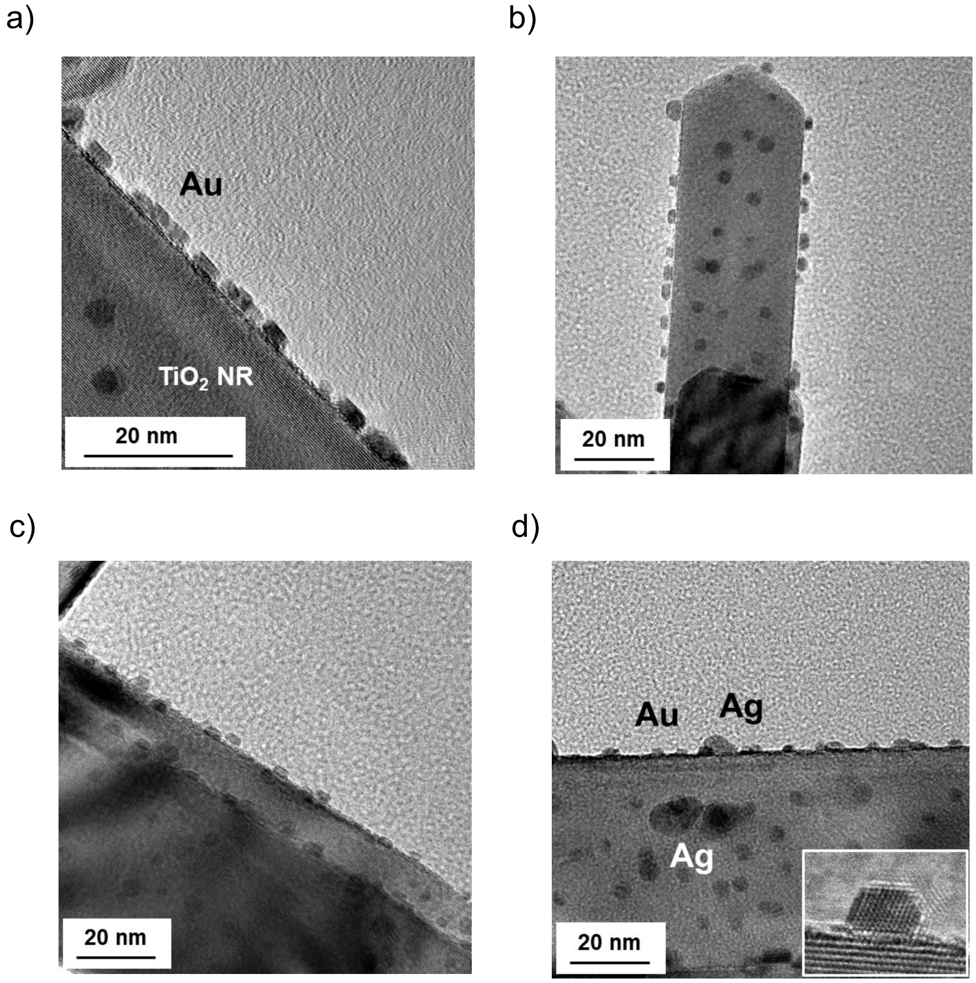

Fig. S13. TEM images of Au/R-TiO_2 NR after reactions: (a) the oxidations of cinnamyl alcohol to cinnamaldehyde, (b) benzylamine to benzaldehyde, (c) the oxidative degradation of 2-naphthol, and (d) O_2 evolution from 10 mM aqueous solution of $AgNO_3$.

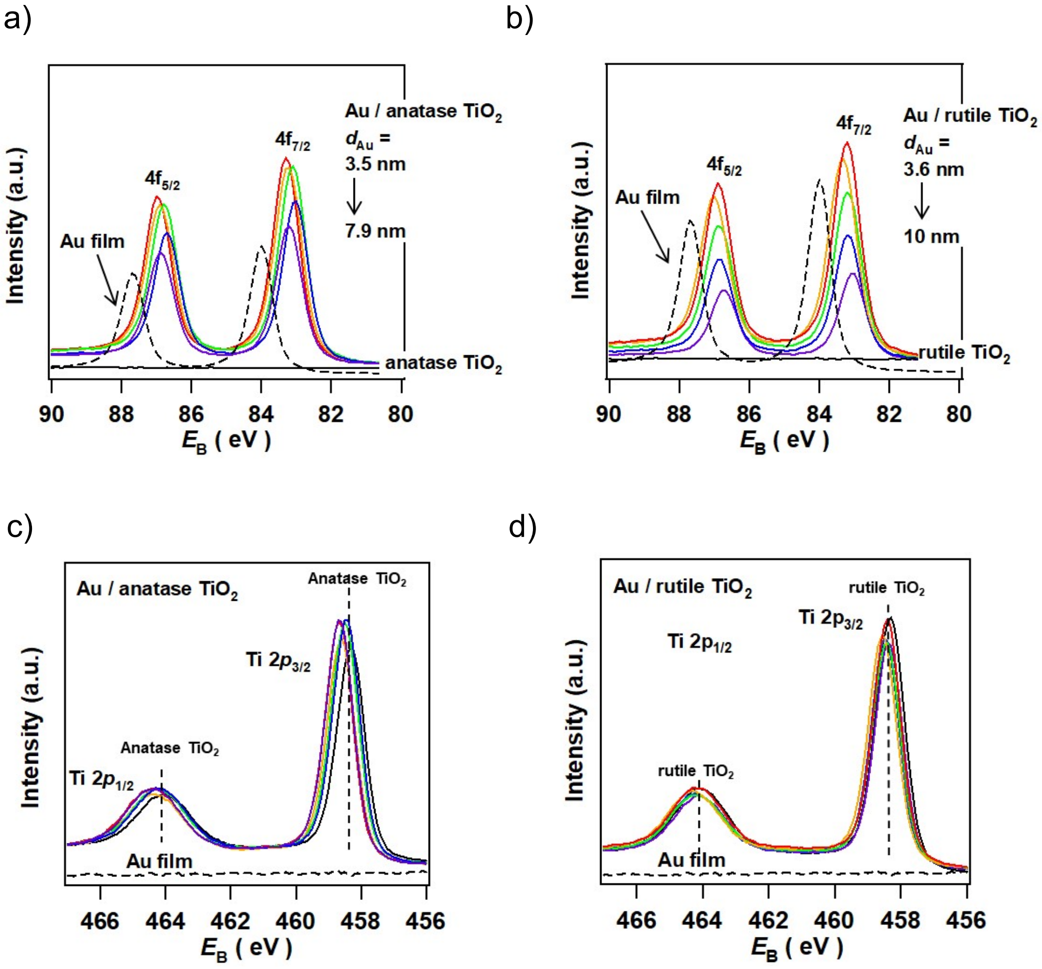

Fig. S14. Au 4f-XP spectra of Au/A-TiO_2 (a) and Au/R-TiO_2 (b) with varying d_{Au}, and unmodified TiO_2 and Au film-coated glass plate for comparison. Ti 2p-XP spectra of Au/A-TiO_2 (c) and Au/R-TiO_2 (d) with varying d_{Au}, and unmodified TiO_2 and Au film-coated glass plate for comparison.

Table S1. Crystal-form effect on the plasmonic photocatalytic activity of Au/TiO_2

Reaction	Reaction rate		ratio	Ref.
	Rutile (R)	Anatase (A)	of R/A	
2-Propanol oxidation	0.545 μmol h^{-1}	0.284 μmol h^{-1}	1.96	S1,2
Cinnamyl alcohol oxidation	6.00 μmol h^{-1}	1.03 μmol h^{-1}	5.81	S3
Benzylamine oxidation	1.82 μmol h^{-1}	0.102 μmol h^{-1}	17.8	S4
H_2 evolution	18.5 μmol h^{-1}	0 μmol h^{-1}	---	S5
Nitrobenzene reduction	7.38 μmol h^{-1}	1.04 μmol h^{-1}	7.10	S6
Oxygen evolution reaction	10.1 μmol h^{-1}	2.24 μmol h^{-1}	4.52	S7
Water oxidation	4.46 μA cm^{-2}	0.109 μA cm^{-2}	40.9	S8
Nonylphenol degradation	2.46 μmol h^{-1}	0.321 μmol h^{-1}	7.65	S9
2-Naphthol degradation	2.40 μmol h^{-1}	0.238 μmol h^{-1}	10.1	S10

Reference

(S1) E. Kowalska, R. Abe and B. Ohtani, Visible light-induced photocatalytic reaction of gold-modified titanium(IV) oxide particles: action spectrum analysis. *Chem. Commun.,* 2009, **45**, 241-243.

(S2) E. Kowalska, O. O. P. Mahaney and B. Ohtani, Visible-light-induced photocatalysis through surface plasmon excitation of gold on titania surfaces. *Phys. Chem. Chem. Phys*., 2010, **12**, 2344-2355.

(S3) K. Kimura, S. Naya, Y. Jin-nouchi and H. Tada, TiO_2 crystal form-dependence of the Au/TiO_2 plasmon photocatalyst's activity. *J. Phys. Chem. C,* 2012, **116**, 7111-7117.

(S4) S. Naya, K. Kimura and H. Tada, One-step selective aerobic oxidation of amines to imines by gold nanoparticle-loaded rutile titanium(IV) oxide plasmon photocatalyst. *ACS Catalysis,* 2013, **3**, 10-13.

(S5) J. B. Priebe, J. Radnik, A. J. J. Lennox, M.-M. Pohl, M. Karnahl, D. Hollmann, K. Grabow, U. Bentrup, H. Junge, M. Beller and A. Bruckner, Solar hydrogen production by plasmonic Au-TiO_2 catalsyt: impact of synthesis protocol and TiO_2 phase on charge transfer efficiency and H_2 evolution rates. *ACS Catal.,* 2015, **5**, 2137-2148.

(S6) S. Naya, T. Niwa, T. Kume and H. Tada, Visible-light-induced electron transport from small to large nanoparticles in bimodal gold nanoparticle-loaded titanium(IV) oxide. *Angew. Chem. Int. Ed.,* 2014, **53** 7305-7309.

(S7) S. Wang, Y. Gao, S. Miao, T. Liu, L. Mu, R. Li, F. Fan and C. Li, Positioning the water oxidation reaction sites in plasmonic photocatalysts. *J. Am. Chem. Soc.,* 2017, **139**, 11771-11778.

(S8) T. Onishi, M. Teranishi, S. Naya, M. Fujishima and H. Tada, Electrocatalytic effect on the photon-to-current conversion efficiency of gold-nanoparticle-loaded titanium(IV) oxide plasmonic electrode for water oxidation. *J. Phys. Chem. C,* 2020*,* **124**, 6103-6109.
(S9) S. Naya, T. Nikawa, K. Kimura and H. Tada, Rapid and complete removal of nonylphenol by gold nanoparticle/rutile titanium(IV) oxide plasmon photocatalyst. *ACS Catal.,* 2013, **3**, 903-907.
(S10) S. Naya and H. Tada, Dependence of the plasmonic activity of Au/TiO_2 for the decomposition of 2-naphthol on the crystal form of TiO_2 and Au particle size. *J. Catal.,* 2018, **364**, 328-333.

Table S2. Characterization results of the Au/A-TiO_2 and Au/R-TiO_2 plasmonic photocatalysts.

Catalysts	d_{Au} / nm (counts)	x_{Au} / mass%	Faceting probability / % (counts)	T_c / K	t_c / h
Au/A-TiO_2	3.5 ± 0.9 (203)	4.25	negligible (1032)	673	1
Au/A-TiO_2	4.1 ± 0.8 (208)	4.20	negligible (1045)	673	4
Au/A-TiO_2	5.0 ± 0.9 (205)	4.21	negligible (1021)	773	4
Au/A-TiO_2	6.6 ± 1.4 (208)	4.24	0.1 (1032)	873	4
Au/A-TiO_2	7.9 ± 1.6 (209)	4.21	0.2 (1037)	873	24
Au/R-TiO_2	3.6 ± 1.1 (210)	4.22	14.1 (1017)	673	1
Au/R-TiO_2	4.0 ± 0.9 (211)	4.16	3.6 (1013)	673	4
Au/R-TiO_2	5.1 ± 1.0 (213)	4.21	1.8 (1009)	773	4
Au/R-TiO_2	7.5 ± 1.9 (213)	4.31	0.9 (1037)	873	4
Au/R-TiO_2	10.0 ± 2.2 (204)	4.19	0.8 (1019)	873	24
Au/R-TiO_2 NR	3.5 ± 0.7 (223)	4.20	94.2 (1029)	673	1
Au/R-TiO_2 NR	6.3 ± 1.1 (205)	4.18	88.7 (1034)	873	24

Table S3. Au 4f and Ti 2p-XP binding energies

Samples	d_{Au} / nm	Au		Ti	
		$4f_{7/2}$ /eV	$4f_{5/2}$ / eV	$2p_{3/2}$ / eV	$2p_{1/2}$ / eV
A-TiO_2	---	---	---	458.3	464.0
Au/A-TiO_2	3.5 ± 0.9	83.3	87.0	458.7	464.4
Au/A-TiO_2	4.1 ± 0.8	83.2	86.9	458.6	464.3
Au/A-TiO_2	5.0 ± 0.9	83.1	86.8	458.5	464.3
Au/A-TiO_2	6.6 ± 1.4	83.0	86.7	458.5	464.3
Au/A-TiO_2	7.9 ± 1.6	83.2	86.9	458.7	464.3
R-TiO_2	---	---	---	458.3	464.2
Au/R-TiO_2	3.6 ± 1.1	83.2	86.9	458.4	464.3
Au/R-TiO_2	4.0 ± 0.9	83.3	87.0	458.6	464.3
Au/R-TiO_2	5.1 ± 1.0	83.2	86.9	458.4	464.3
Au/R-TiO_2	7.5 ± 1.9	83.2	86.9	458.5	464.2
Au/R-TiO_2	10.0 ± 2.2	83.1	86.7	458.4	464.2

Table S4. Comparison of the local electric field between HS Au/A-TiO_2 and t-Oh Au/R-TiO_2 systems calculated by the 3D-FDTD method

Model	d_{Au} / nm	λ / nm	Monitor plane	Maximum E / E_0	Maximum $\|E\|^2$ / $\|E_0\|^2$
HS Au/A-TiO_2	3.5	702	*xz*	49.6	2.5E+03
			xy	59.9	3.6E+03
t-Oh Au/R-TiO_2	3.6	675	*xz*	138	1.9E+04
			xy	382	1.5E+05
		704	*xz*	272	7.4E+04
			xy	453	2.1E+05
		722	*xz*	448	2.0E+05
			xy	448	2.0E+05
